# Hypertrophic cardiomyopathy: comprehensive insights into pathogenic genes and genotype-phenotype associations

**DOI:** 10.3389/fcell.2026.1741252

**Published:** 2026-01-30

**Authors:** Luwen Hao, Xin Chen, Bo Qin

**Affiliations:** 1 Department of Radiology, Taikang Tongji (Wuhan) Hospital, Wuhan, China; 2 Department of Cardiovascular Surgery, Shenzhen Qianhai Taikang Hospital, Shenzhen, China; 3 Department of Cardiovascular Surgery, Taikang Tongji (Wuhan) Hospital, Wuhan, China

**Keywords:** genotype–phenotype correlation, hypertrophic cardiomyopathy, MYBPC3, MYH7, sarcomeric genes

## Abstract

Hypertrophic cardiomyopathy (HCM) is a genetically heterogeneous cardiac disorder characterized by unexplained left ventricular hypertrophy and represents a leading cause of morbidity and sudden cardiac death, particularly in young adults and athletes. Early studies focused on morphological features, but advances in molecular genetics have shifted emphasis toward genetic diagnosis, mechanistic insights, and family-based management. Pathogenic variants in sarcomeric genes, especially *MYBPC3* and *MYH7*, are central to disease development, with specific mutation types linked to distinct hypertrophy patterns and clinical outcomes. The phenotype is further modulated by ethnicity, age, and sex, contributing to substantial variability. Implementation of genetic testing has enabled identification of definitive pathogenic variants, highlighting the critical role of genomics in diagnosis and personalized care. Despite progress, challenges remain in interpreting variants of uncertain significance, defining genotype–phenotype correlations, and developing robust risk stratification models and individualized therapeutic strategies. This review summarizes current evidence on the pathogenic gene spectrum, genotype–phenotype correlations, and ethnic- or sex-based variability in HCM, as well as the gene and phenotypic characteristics of pediatric HCM, providing a comprehensive framework for understanding its molecular diversity and guiding precision diagnosis and management.

## 1 Introduction

Hypertrophic cardiomyopathy (HCM) is a common genetic cardiac disorder characterized by unexplained left ventricular hypertrophy (LVH), which can lead to heart failure, arrhythmias, and sudden cardiac death (SCD), particularly in young individuals (Argirò et al., 2025; Marian, 2021). The prevalence of HCM, when defined by the presence of LVH, is estimated to be between 1:300 and 1:600. However, when genetic testing and family screening are incorporated, the estimated prevalence reaches approximately 1:250 in the general population (Marian, 2021). Clinical presentation is markedly heterogeneous, ranging from lifelong asymptomatic status to severe complications such as heart failure, atrial fibrillation, embolic stroke, and SCD—a spectrum that has long posed substantial challenges for clinical management (Argirò et al., 2025; Marian, 2021; Sikand et al., 2025).

The genetic basis of HCM was first established over 3 decades ago with the identification of a missense mutation in the β-cardiac myosin heavy chain gene, defining HCM as a “disease of the sarcomere” (Geisterfer-Lowrance et al., 1990; Calore et al., 2025). Nevertheless, pathogenic variants in genes encoding cardiac sarcomeric proteins are detected in only 30%–40% of cases, while more than 50% of clinically diagnosed HCM patients lack identifiable sarcomeric mutations (Lopes et al., 2024). More than 20 genes have been implicated in HCM, including *MYBPC3*, *MYH7*, *TNNT2*, *TNNI3*, *TPM1*, *MYL2*, *MYL3*, *ACTC1*, *TNNC1*, *ACTN2*, *ALPK3*, and *FHOD3*, which exhibit autosomal dominant inheritance with variable penetrance and diverse expression (Choi et al., 2025). Among these, the first eight sarcomeric genes remain the most strongly associated, accounting for over 90% of genotype-positive cases (Lopes et al., 2024; Choi et al., 2025; Ingles et al., 2019; Tadros et al., 2025).

Clinical manifestations of HCM arise from complex interactions between genetic and non-genetic factors, with genetic architecture playing a predominant role in shaping phenotypic heterogeneity (Marian, 2021). The specific gene involved, the type and location of the mutation, and the presence of multiple variants—such as compound heterozygosity—significantly influence age of onset, extent of hypertrophy, risk of left ventricular outflow tract obstruction, and overall prognosis (Marian, 2021; Lopes et al., 2024; Wang et al., 2025; Maron et al., 2022). Therefore, delineating genotype–phenotype correlations is not only academically relevant but also a clinical imperative, forming the basis for early diagnosis, improved risk stratification, cascade screening of at-risk relatives, and the development of individualized therapeutic strategies. This narrative review integrates current advances in the genetic basis and phenotypic features of HCM, with a focus on the mutational spectrum and clinical implications of key pathogenic genes. It also summarizes the genetic and phenotypic characteristics of pediatric HCM and examines the influence of ethnicity and sex on HCM phenotypes.

## 2 Methodology

A comprehensive search was conducted in PubMed and Web of Science, supplemented by hand-searching the reference lists of key publications. The search focused on studies published between 1990 and 2025, using combinations of terms such as “Hypertrophic cardiomyopathy”, “gene”, “genotype–phenotype”, “pathogenesis”, and “clinical management”. Both original research and review papers were considered if they were relevant to our research question. Inclusion criteria were: Clinical or mechanistic relevance; High methodological quality; and contribution to understanding genetic spectrum, genotype–phenotype correlations, or clinical implications. Studies with insufficient methodological clarity, duplicated data, or lacking relevance were excluded.

## 3 Epidemiology of pathogenic genes in HCM

Among genes implicated in HCM, *MYBPC3* and *MYH7* are the two most prevalent, together accounting for approximately 70%–80% of genetically confirmed cases and about 35% of all clinically diagnosed HCM cases (∼20% for *MYBPC3*, ∼15% for *MYH7*) (Chiswell et al., 2023). *MYBPC3* encodes cardiac myosin-binding protein C, while *MYH7* encodes the β-myosin heavy chain; both are critical components of the thick filament in the cardiac sarcomere, essential for maintaining contractile integrity. Remaining pathogenic variants primarily involve other thick filament genes (e.g., *MYL2*, *MYL3*), thin filament proteins (e.g., *TNNT2*, *TNNI3*, *TNNC1*, *ACTC1*), and Z-disc or cytoskeletal components (e.g., *MYOZ2*, *ACTN2*, *TCAP*). These genes collectively contribute to sarcomere structure, contractile regulation, calcium sensitivity, and mechanosensory signaling (Choi et al., 2025; Walsh et al., 2017; García-Hernández et al., 2024).

Mutations in troponin complex genes (e.g., *TNNT2*, *TNNI3*, *TNNC1*) are detected in approximately 10% of genotype-positive cases, while mutations in *ACTC1* and myosin light chain genes (*MYL2*, *MYL3*) collectively account for less than 5%–10%. Overall, pathogenic sarcomeric variants explain roughly 60%–70% of familial HCM cases (Choi et al., 2025).

**FIGURE 1:**
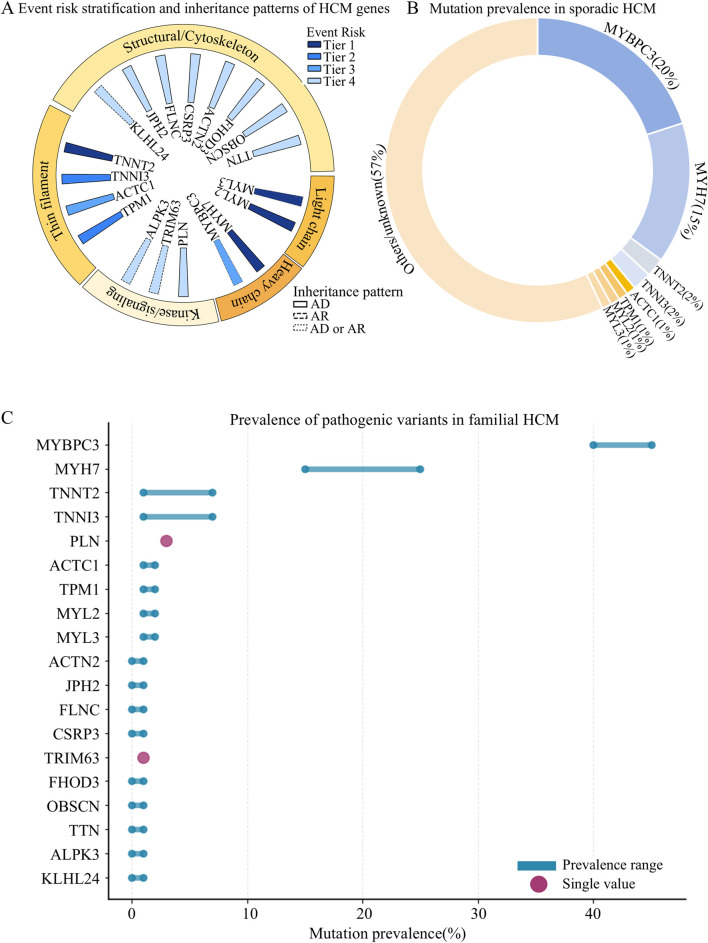
Epidemiological characteristics of pathogenic genes in HCM. **(A)** Event risk stratification and inheritance patterns of HCM genes; **(B)** Mutation prevalence in sporadic HCM; **(C)** Prevalence of pathogenic variants in familial HCM.

Individuals carrying pathogenic or likely pathogenic sarcomeric mutations tend to develop HCM earlier, exhibit higher penetrance, and face a two-fold greater risk of arrhythmias, SCD, and other adverse cardiovascular events compared with mutation-negative individuals (Nakashima et al., 2020; Ho et al., 2018). Here, we summarized the epidemiological characteristics of pathogenic genes in HCM (Supplementary Table S1; Figure 1).

## 4 The major two heavy chain genes

### 4.1 *MYBPC3* mutations and pathogenic mechanisms

The *MYBPC3* gene encodes cardiac myosin-binding protein C (cMyBP-C), a sarcomeric protein essential for sarcomere integrity and regulation of cardiac contraction and relaxation (Tudurachi et al., 2023). It is the most frequently mutated gene in HCM, accounting for approximately 20% of cases (Sedaghat-Hamedani et al., 2018). Truncating mutations—including frameshift, nonsense, and splice-site variants that introduce premature termination codons—constitute about 50% of *MYBPC3* variants (Tudurachi et al., 2023). These typically lead to haploinsufficiency via nonsense-mediated decay and proteasomal degradation, reducing myocardial cMyBP-C levels by 30%–50%. The resulting stoichiometric imbalance and loss of super-relaxed myosin lead to hypercontractility, increased ATP consumption, and impaired relaxation (Suay-Corredera et al., 2021; Andersen et al., 2004). In rare cases, truncated proteins escape degradation and exert a dominant-negative “poison peptide” effect, exacerbating hypertrophy and fibrosis (Marian, 2021; Tudurachi et al., 2023; Kuster et al., 2019; Kuster et al., 2015).

Non-truncating variants (∼15%), including missense and in-frame indels, often reduce protein stability or disrupt interactions with myosin and actin, altering cross-bridge kinetics and increasing energy demand (Tudurachi et al., 2023; Helms et al., 2020; Page et al., 2012). Their clinical manifestations largely mirror those of truncating variants. *MYBPC3* mutations are frequently associated with mid-septal hypertrophy in Han Chinese patients (Zhou et al., 2023), and carriers exhibit greater interventricular septal thickness but later onset (mean > 35 years) than *MYH7* carriers (Sedaghat-Hamedani et al., 2018). A male predominance has been observed (Sedaghat-Hamedani et al., 2018). The p. Val158Met variant has been linked to SCD, particularly in combination with *TNNT2* p. Lys263Arg or *MYH7* p. Val320Met (Mori et al., 2021).

### 4.2 Clinical features and penetrance

*MYBPC3*-related HCM shows an estimated penetrance of ∼55%, with affected individuals often developing ventricular arrhythmias or syncope but generally maintaining a favorable prognosis (Topriceanu et al., 2024; Chida et al., 2017; Abbas et al., 2024). The heart transplantation rate among *MYBPC3* carriers is low (0.6%) compared with *MYH7*-positive patients (7.7%) (Sedaghat-Hamedani et al., 2018). Homozygous or compound heterozygous truncating variants can cause severe neonatal cardiomyopathy with high mortality, while double heterozygotes exhibit more aggressive phenotypes than single heterozygotes (Chida et al., 2017; Carrier, 2021).

### 4.3 *MYH7* mutations and pathogenic mechanisms

The *MYH7* gene encodes the β-myosin heavy chain, the major motor protein of the cardiac sarcomere, and is the second most commonly mutated gene in HCM, accounting for ∼15% of cases (Sedaghat-Hamedani et al., 2018). Over 200 pathogenic variants—primarily missense mutations—cluster in the motor and converter domains, regions critical for ATPase activity and mechanical transduction (Gao et al., 2024; Homburger et al., 2016; García-Giustiniani et al., 2015). These mutations impair cross-bridge kinetics, delay relaxation, and increase myocardial energy expenditure (Lopes et al., 2019; Toepfer et al., 2020).

Cellularly, stochastic transcriptional “bursting” causes allelic imbalance between wild-type and mutant *MYH7* mRNA, leading to cell-to-cell contractile heterogeneity and variable hypertrophy (Tripathi et al., 2011; Montag et al., 2017; Montag et al., 2018). Certain mutations (e.g., p. R723G) destabilize transcripts, amplifying this imbalance (Rose et al., 2020). Phenotypic heterogeneity is notable even among monozygotic twins carrying identical mutations (e.g., p. G768R) (Wang J. et al., 2019).

Compound heterozygous or double *MYH7* mutations are associated with earlier onset, more severe hypertrophy, and worse prognosis, supporting a dose-dependent effect (Wang B. et al., 2019; Zhang et al., 2022; Wang et al., 2017; Wang et al., 2016; Rodríguez-López et al., 2022; Dorn and McNally, 2014; Blankenburg et al., 2014; Richard et al., 2000). The I736T variant significantly destabilizes β-myosin and compromises structural stability (Ahmad et al., 2022), while p. Val320Met increases SCD risk (Mori et al., 2021).

### 4.4 Clinical phenotypes and comparison with *MYBPC3*

*MYH7*-related HCM clinically manifests with asymmetric septal hypertrophy—often involving the anterior wall, interventricular septum, and lateral wall—and a higher septum-to-posterior wall ratio than *MYBPC3*-related disease (Sedaghat-Hamedani et al., 2018; Zhou et al., 2023). This genotype also confers risk for dilated cardiomyopathy, left ventricular noncompaction, Ebstein anomaly, and skeletal myopathy (Postma et al., 2011), alongside higher rates of ventricular tachycardia, atrioventricular block, and bundle branch block compared to *MYBPC3* (Sedaghat-Hamedani et al., 2018). *MYH7* further distinguishes itself by having the highest penetrance (∼65%) among major genes (*cf. MYBPC3*: ∼55%; *TNNT2*/*TNNI3*: ∼60%; *MYL3*: ∼32%) (Topriceanu et al., 2024) and the earliest mean age of onset (35 years), preceding *MYBPC3*/*TNNT2* (39 years) and TNNI3/mutation-negative groups (44 years) (Sedaghat-Hamedani et al., 2018).

According to SHaRe registry data, *MYH7* carriers face greater risks of atrial fibrillation, advanced heart failure, and transplantation (Ho et al., 2018; Sedaghat-Hamedani et al., 2018). No consistent sex bias is observed (Sedaghat-Hamedani et al., 2018), but the overall phenotype is more aggressive, with earlier onset, increased arrhythmogenicity, and faster progression to systolic dysfunction. In contrast, *MYBPC3* carriers present later, with dyspnea and strong familial aggregation but milder structural changes (Velicki et al., 2020).

Longitudinal imaging shows no significant differences in left or right ventricular strain between *MYBPC3* and *MYH7* carriers (Höller et al., 2021). However, patients with either genotype undergoing atrial fibrillation ablation exhibit more low-amplitude left atrial signals, suggesting greater fibrosis, though ablation remains effective (Haq et al., 2024). Despite similar outcomes, *MYBPC3*-related HCM shows a higher long-term prevalence of systolic dysfunction, indicating distinct progression mechanisms between genotypes (Beltrami et al., 2023) (Table 1).

**TABLE 1:** Clinical features and phenotypic characteristics of *MYBPC3* and *MYH7* mutations.

Feature	*MYBPC3* mutations	*MYH7* mutations
Prevalence	• ∼20% of HCM cases (Chiswell et al., 2023; Sedaghat-Hamedani et al., 2018)	• ∼15% of HCM cases (Sedaghat-Hamedani et al., 2018)
Mutation types	• Truncating (50%), missense, in-frame indels (Tudurachi et al., 2023)	• Primarily missense, clustering in motor/converter domains (Gao et al., 2024; Homburger et al., 2016; García-Giustiniani et al., 2015)
Major cardiac complications	• Ventricular arrhythmias and syncope are commonly observed (Topriceanu et al., 2024, Chida et al., 2017) • Overall prognosis for heart failure is relatively favorable (Topriceanu et al., 2024; Chida et al., 2017) • Heart transplantation rate is low (0.6%) (Sedaghat-Hamedani et al., 2018) • Higher long-term prevalence of systolic dysfunction (Beltrami et al., 2023)	• Higher risk of arrhythmias, including ventricular tachycardia, atrioventricular block, bundle branch block (Sedaghat-Hamedani et al., 2018), and atrial fibrillation (Ho et al., 2018; Sedaghat-Hamedani et al., 2018) • Higher risk of advanced heart failure (Ho et al., 2018; Sedaghat-Hamedani et al., 2018) • Heart transplantation rate is significantly higher (7.7%) (Sedaghat-Hamedani et al., 2018)
Structural complications /Phenotypes	• Frequently presents as mid-septal hypertrophy in han Chinese patients (Zhou et al., 2023) • Structural changes are generally milder (Velicki et al., 2020)	• More typical asymmetric septal hypertrophy, often involving the anterior wall, interventricular septum, and lateral wall, with a higher septum-to-posterior wall ratio (Sedaghat-Hamedani et al., 2018; Zhou et al., 2023) • Broader phenotypic spectrum, which may include: Dilated cardiomyopathy, left ventricular noncompaction, ebstein anomaly (Postma et al., 2011)
Other related complications	• SCD risk: The p.Val158Met variant increases risk, particularly when combined with *TNNT2* p.Lys263Arg or *MYH7* p.Val320Met variants (Mori et al., 2021)	• SCD risk: The p.Val320Met variant increases risk (Mori et al., 2021) • Skeletal myopathy (present in some cases) (Postma et al., 2011)
Disease course and features	• Later onset (mean 39 years) [(Sedaghat-Hamedani et al., 2018) • Moderate penetrance (∼55%) (Topriceanu et al., 2024) • Male predominance observed (Sedaghat-Hamedani et al., 2018) • Familial aggregation (Velicki et al., 2020)	• Earliest onset (mean 35 years) (Sedaghat-Hamedani et al., 2018) • Highest penetrance (∼65%) (Topriceanu et al., 2024) • No consistent sex bias (Sedaghat-Hamedani et al., 2018) • More aggressive overall phenotype with faster progression to systolic dysfunction (Velicki et al., 2020)

## 5 The other heavy chain genes

The *MYL2* and *MYL3* genes encode the regulatory and essential myosin light chains, which fine-tune myosin ATPase activity and cross-bridge cycling. Pathogenic variants are rare, accounting for ∼1% of HCM cases (Blankenburg et al., 2014; Kabaeva et al., 2002), and are typically associated with apical or atypical hypertrophy, sometimes with familial inheritance. Although uncommon, these variants confer adverse outcomes and more severe progression than *MYBPC3* or *MYH7* mutations (Ahmad et al., 2022). Mechanistically, altered light chain phosphorylation affects sarcomeric energetics and contractile efficiency, amplifying disease severity.

## 6 Thin filament genes

Mutations in *TNNT2*, *TNNI3*, *ACTC1*, and *TPM1* disrupt calcium-dependent regulation of actin–myosin interaction, contributing to diverse HCM phenotypes.

### 6.1 *TNNT2* (cardiac troponin T)

Accounting for ∼2% of HCM cases (Sedaghat-Hamedani et al., 2018), *TNNT2* variants (mostly missense) anchor the troponin–tropomyosin complex to actin. They often cause mild hypertrophy but disproportionate myocyte disarray, predisposing to malignant arrhythmias (Pasquale et al., 2012; McKenna et al., 1990; Varnava et al., 2000; Shakur et al., 2021). In Han Chinese patients, *TNNT2* mutations are associated with anterior and septal hypertrophy (Zhou et al., 2023). The p. Lys263Arg variant markedly increases SCD risk, especially when combined with *MYBPC3* or *MYH7* variants (Mori et al., 2021). A male predominance has been reported (Sedaghat-Hamedani et al., 2018).

### 6.2 *TNNI3* (cardiac troponin I)

*TNNI3* mutations contribute to ∼2% of HCM cases (Sedaghat-Hamedani et al., 2018) and cluster in residues 131–176, corresponding to the troponin C-binding region (Kubo et al., 2007; Muchtar et al., 2017; Mogensen et al., 2003). They typically cause anterior and septal hypertrophy (Zhou et al., 2023). Carriers may experience atrial arrhythmias, though statistical significance is limited (Sedaghat-Hamedani et al., 2018).

### 6.3 *ACTC1* (cardiac α-actin) and *TPM1* (α-tropomyosin)

*ACTC1* mutations (∼1% of cases) affect early sarcomerogenesis and may cause overlapping phenotypes such as noncompaction cardiomyopathy or septal defects (Monserrat et al., 2007; Despond and Dawson, 2018). *TPM1* mutations (∼1%) are characterized by anterior/septal hypertrophy (Zhou et al., 2023), pronounced phenotypic variability, and often worse cardiac outcomes than *MYH7* or *MYBPC3* mutation groups (Ahmad et al., 2022). The K233N variant is structurally deleterious, disrupting actin–myosin regulation (Ahmad et al., 2022).

## 7 Z-disc and cytoskeletal genes

Beyond thick and thin filament proteins, several Z-disc and cytoskeletal genes act as established contributors or modifiers of HCM. *ALPK3* variants cause a distinct apical hypertrophy pattern (Zhou et al., 2023; Phelan et al., 2016; Almomani et al., 2016; Cheawsamoot et al., 2020; Lopes et al., 2021), defining a morphologically unique HCM subtype. *TTN* and *OBSCN* mutations lead to diffuse or uniform myocardial hypertrophy, implicating cytoskeletal disruption rather than classical sarcomeric dysfunction (Zhou et al., 2023; Saha et al., 2025; Wu et al., 2021). Other rare but relevant genes include *FHOD3* (Ochoa et al., 2018), *ACTN2* (Bagnall et al., 2014; Prondzynski et al., 2019; Noureddine et al., 2025), *TRIM63* (Salazar-Mendiguchía et al., 2020a; Bonanni et al., 2026), *PLN* (Ceholski et al., 2012; Young et al., 2015; van Drie et al., 2025), *CSRP3* (Geier et al., 2008; Salazar-Mendiguchía et al., 2020b), *FLNC* (Valdés-Mas et al., 2014; Verdonschot et al., 2020; Cui et al., 2018), *JPH2* (Parker et al., 2023; Landstrom et al., 2007), and *KLHL24* (Hedberg-Oldfors et al., 2019), each affecting sarcomeric architecture, calcium homeostasis, or mechanotransduction in specific contexts.

## 8 Genotype-negative and double-mutant HCM

Patients without detectable sarcomeric mutations often show greater left ventricular outflow tract (LVOT) obstruction despite less hypertrophy (Sedaghat-Hamedani et al., 2018). Interestingly, carriers of double or compound mutations do not consistently exhibit more severe phenotypes than single-variant or genotype-negative patients, suggesting potential epistatic or compensatory interactions (Ahmad et al., 2022).

## 9 Characteristics of pediatric hypertrophic cardiomyopathy (PHCM)

Pediatric hypertrophic cardiomyopathy (PHCM) is the second most frequent primary myocardial disorder in children and adolescents and a leading cause of sudden cardiac death among young athletes. Unlike adult-onset HCM, which often presents as isolated LVH, PHCM exhibits greater phenotypic and etiological heterogeneity. Underlying causes include inborn errors of metabolism, neuromuscular and malformation syndromes, and genetic mutations affecting sarcomeric proteins—the latter accounting for most apparently idiopathic cases (Moak and Kaski, 2012).

In pediatric cohorts, pathogenic variants in *MYBPC3* and *MYH7* are the predominant molecular causes, similar to adults (Darwish et al., 2020). Approximately 63.6% of affected children carry a detectable pathogenic or likely pathogenic variant, a rate higher than in adults (Nguyen et al., 2022). Importantly, children with a positive genetic result are more likely to exhibit extracardiac manifestations (38.1% vs. 8.3%) and have increased clinical severity, reflected by higher rates of implantable cardioverter-defibrillator implantation (23.8% vs. 0%) and heart transplantation (19.1% vs. 0%) (Wanert et al., 2023). These findings indicate that genetic status influences not only disease onset but also progression and prognosis in PHCM.

Growing evidence supports a strong relationship between genotype, diastolic function, and clinical outcomes in pediatric HCM(90). Specific allelic variants, such as the VEGF1 963 GG allele, have been associated with reduced left ventricular systolic and diastolic performance (Pieles et al., 2021), suggesting that subtle genetic modifiers may influence myocardial mechanics even in the absence of classical sarcomere mutations. Furthermore, diffuse interstitial fibrosis is common in pediatric patients and likely underrecognized, though its association with long-term outcomes remains inadequately characterized (Nguyen et al., 2022; Hussain et al., 2015).

From a molecular standpoint, *MYH7* variants play a pivotal role in early-onset disease. Missense mutations such as p. R719W, p. R453C, and p. Y386C have been linked to a spectrum of presentations, from non-obstructive and restrictive phenotypes to severe conduction defects and SCD (Vasilescu et al., 2018; Mathew et al., 2018; Bobkowsk et al., 2007; Greenway et al., 2012). Additionally, *MYH7*-related congenital heart diseases (CHD) in children frequently co-occurs with structural anomalies including ventricular septal defect, Ebstein anomaly, hypoplastic left heart syndrome, double-outlet right ventricle, left ventricular noncompaction, and arrhythmias (Kuroda et al., 2022). Collectively, these observations emphasize that *MYH7*-associated pediatric cardiomyopathy exhibits greater clinical heterogeneity and more aggressive progression than adult-onset cases. Regarding disease onset, metabolic and syndromic forms typically present in infancy or early childhood, while neuromuscular-related HCM often appears in adolescence (Moak and Kaski, 2012). Infants are commonly identified during evaluation for a cardiac murmur or heart failure symptoms, whereas older children may come to attention due to abnormal electrocardiogram (ECG) findings, exertional intolerance, or family screening.

PHCM that persists into adulthood is predominantly driven by pathogenic sarcomeric gene mutations acting in an autosomal dominant manner, most commonly involving MYH7 and MYBPC3(88–91). These pathogenic variants constitute the primary disease-causing determinants and are sufficient to initiate early myocardial hypertrophy, diastolic dysfunction, and progressive remodeling. However, the clinical manifestation of these mutations is often age-dependent and influenced by incomplete penetrance and variable expressivity, which may explain why some individuals carrying pathogenic variants do not exhibit overt hypertrophy or symptoms during childhood and only present with HCM in adulthood. In contrast, non-mutagenic or modifier genes do not independently cause PHCM but may synergistically modulate phenotypic expression, disease severity, and clinical trajectory. Evidence indicates that genetic modifiers, such as specific allelic variants including VEGF1 963 GG, can influence myocardial systolic and diastolic performance even in the absence of classical sarcomeric mutations, suggesting a role in modifying disease penetrance and progression (Pieles et al., 2021). Furthermore, children carrying pathogenic sarcomeric mutations demonstrate higher rates of extracardiac involvement, adverse clinical outcomes, and need for advanced interventions compared with genotype-negative patients, underscoring the dominant contribution of pathogenic mutations to disease severity and prognosis (Wanert et al., 2023). Recessive and dominant inheritance patterns are mainly observed in metabolic or syndromic cardiomyopathies presenting in infancy or early childhood and rarely account for PHCM cases that persist into adulthood (Moak and Kaski, 2012; Monda et al., 2021).

Risk stratification in PHCM remains a major clinical challenge. Heterogeneous genetic background, variable expressivity, and age-dependent penetrance complicate risk prediction. Therefore, comprehensive family-based genetic evaluation—including screening of first-degree relatives and at-risk family members—is strongly recommended, given the predominance of familial aggregation in pediatric cases (Moak and Kaski, 2012; Monda et al., 2021). Early identification of high-risk genotypes, especially in *MYH7* or *MYBPC3*, is essential for timely intervention and tailored management (Table 2).

**TABLE 2:** Characteristics of PHCM.

Category	Key points
Overview	• Second most common childhood myocardial disorder • Leading cause of SCD in young athletes • More heterogeneous than adult HCM (Moak and Kaski, 2012)
Genetics	• Predominant mutations in *MYBPC3* and *MYH7* • ∼63.6% have a pathogenic variant • Genetic positive cases have more extracardiac issues and severe outcomes (ICD, transplant) (Darwish et al., 2020; Nguyen et al., 2022; Wanert et al., 2023)
Genotype-phenotype	• Genotype links to diastolic function and outcomes • Specific alleles (e.g., *VEGF1* 963 GG) impair ventricular function • Diffuse fibrosis is common (Nguyen et al., 2022; Pieles et al., 2021; Hussain et al., 2015)
*MYH7* variants	• Crucial for early-onset disease • Missense mutations cause diverse phenotypes (non-obstructive, restrictive, conduction defects, SCD) • Often co-occurs with CHD (Vasilescu et al., 2018; Mathew et al., 2018; Bobkowsk et al., 2007; Greenway et al., 2012; Kuroda et al., 2022)
Disease onset	• Metabolic/Syndromic: Infancy/Early childhood • Neuromuscular: Adolescence • Presentation: Murmur/HF (infants), abnormal ECG/exertional intolerance/family screening (older) (Moak and Kaski, 2012)
Risk and management	• Risk stratification is challenging • Comprehensive family genetic screening is recommended • Early identification of high-risk genotypes is key for management (Moak and Kaski, 2012)

## 10 Ethnic and regional differences

### 10.1 Comparasion between Asian and European

Comparative analyses between Asian and European centers reveal distinct demographic, clinical, and therapeutic patterns. Asian patients are typically diagnosed at an older age (median 59 vs. 52 years), have smaller body surface area, and exhibit a higher prevalence of hypertension and coronary artery disease than their European counterparts (Tjahjadi et al., 2022). Morphologically, apical hypertrophy is the predominant subtype in Asian patients (31%), whereas septal hypertrophy with LVOT obstruction is more frequent in Europeans (28%).

Genetic testing practices differ markedly: only 3% of Asian patients underwent genotyping versus 17% in European centers (Tjahjadi et al., 2022). This disparity likely reflects differences in healthcare accessibility, testing cost, and genetic counseling awareness in Asia. Moreover, β-blockers were prescribed more frequently in European centers (61% vs. 49%), while calcium channel blockers were more common in Asia (25% vs. 16%), possibly due to lower β-blocker tolerance and reduced obstructive HCM prevalence in Asian populations (Table 3).

**TABLE 3:** Ethnic differences in HCM (Asian vs. European cohorts) (Tjahjadi et al., 2022).

Feature	Asian patients	European patients	Key takeaways
Age at diagnosis	Older (median 59 years)	Younger (median 52 years)	Asian patients are typically diagnosed at an older age
Body surface area	Smaller	Larger	Asian patients have a generally smaller physique
Comorbidities	Higher prevalence of hypertension and coronary artery disease	Lower prevalence of hypertension and coronary artery disease	Asian patients have a higher burden of cardiovascular risk factors
Hypertrophy pattern	Apical hypertrophy (31%)	Septal hypertrophy with LVOT obstruction (28%)	There are significant regional differences in disease expression
Genetic testing	3% undergo genotyping	17% undergo genotyping	Genetic testing is much more common in europe, reflecting differences in healthcare access and awareness
Treatment patterns	Calcium channel blockers are more common	β-blockers are prescribed more frequently	Treatment choices are influenced by disease subtype prevalence and patient tolerance

### 10.2 The other countries/regions/ethnicities

In the Indian (n = 30) and Brazilian (n = 55) cohorts, *MYBPC3* was identified as the predominant disease-associated gene (Mori et al., 2021; Ahmad et al., 2022). In the Icelandic population (n = 180), the founder mutation *MYBPC3* c.927–2A>G was the predominant pathogenic factor, accounting for approximately 58% of cases (Adalsteinsdottir et al., 2014). In contrast, mutations in genes such as *MYH7* and *TPM1* also played a significant role in Japanese (n = 211), Vietnamese (n = 104), and South African (n = 43) populations (Nakashima et al., 2020; Tran et al., 2019; Ntusi et al., 2016). Notably, a multi-ethnic U.S. study (n = 602) demonstrated substantial racial disparities in the detection rates of pathogenic or likely pathogenic variants, with the highest rate observed in Asian patients (65%) and the lowest in African ancestry patients (24%) (Gal et al., 2022).

In terms of clinical phenotypes and prognosis, population-specific characteristics are equally evident. Among Japanese patients, carriers of sarcomeric gene mutations presented with earlier disease onset, more pronounced interventricular septal hypertrophy, and a significantly higher lifetime risk of HCM-related adverse events (Nakashima et al., 2020). Another Japanese study (n = 140) indicated that *MYBPC3* mutations were associated with arrhythmias and syncope (Chida et al., 2017). In the Brazilian cohort, specific variants (*MYBPC3* p. Val158Met and *TNNT2* p. Lys263Arg) were linked to severe left ventricular hypertrophy (Mori et al., 2021). Data from Finnish (n = 382) and Icelandic populations showed that carriers of pathogenic mutations had higher rates of implantable cardioverter-defibrillator implantation and adverse events, with HCM-related mortality occurring significantly earlier (Adalsteinsdottir et al., 2014; Jääskeläinen et al., 2019). Additionally, a high rate of consanguinity (62.5%) was observed among Egyptian pediatric patients (n = 24), suggesting a distinct genetic background in this population (Darwish et al., 2020).

The genetic basis and clinical manifestations of HCM exhibit marked population specificity. These findings underscore the necessity of integrating population-specific epidemiological and genetic characteristics into risk stratification and clinical management to achieve individualized and precise patient care.

## 11 Sex-based differences in clinical expression and outcomes

Sex-specific differences represent another major dimension of phenotypic variability in HCM. Women are generally diagnosed at an older age and have smaller left ventricular volumes but worse diastolic function, often presenting with more severe symptoms such as exertional dyspnea, fatigue, and limited exercise capacity (Constantine et al., 2020; Bonaventura et al., 2021). Despite less pronounced hypertrophy, women frequently exhibit obstructive physiology, leading to a higher incidence of heart failure symptoms and poorer clinical outcomes.

In contrast, men tend to present earlier in life, with greater ventricular mass, wall thickness, and cavity dilation, suggesting sex-linked differences in cardiac remodeling. Hormonal factors, particularly estrogen and androgen signaling, may modulate myocardial fibrosis and calcium handling, contributing to these divergent phenotypes (Constantine et al., 2020). *TNNI3* mutations are more frequent in females, whereas *MYBPC3* and *TNNT2* mutations, as well as mutation-negative cases, are more common in males (Sedaghat-Hamedani et al., 2018). Notably, even after adjusting for genotype, female sex remains an independent predictor of increased mortality and progression to advanced heart failure, underscoring its impact on long-term myocardial performance (Chou and Chin, 2021) (Table 4).

**TABLE 4:** Sex-based differences in HCM clinical expression (Sedaghat-Hamedani et al., 2018; Constantine et al., 2020; Bonaventura et al., 2021; Chou and Chin, 2021).

Feature	Female	Male
Age at diagnosis	Older	Younger
Cardiac structure and function	Smaller LV volumes, worse diastolic function	Greater ventricular mass and wall thickness, often with cavity dilation
Symptom severity	More severe (exertional dyspnea, fatigue, limited exercise capacity)	Less severe
Hypertrophy vs. obstruction	Less hypertrophy, but more frequent obstructive physiology	More pronounced hypertrophy
Clinical outcomes	Poorer (higher HF incidence and mortality)	Relatively better
Underlying mechanism	Sex hormones (e.g., estrogen/Androgen) signaling collectively contribute to divergent cardiac remodeling phenotypes	

## 12 Genetic heterogeneity and phenotypic modifiers

HCM is characterized by marked genetic heterogeneity, with over 1,500 known pathogenic or likely pathogenic variants identified in more than 30 sarcomeric and related genes. However, clear one-to-one correlations between specific mutations and phenotypes remain limited, as many variants are “private” and demonstrate variable penetrance among individuals and families (Bonaventura et al., 2021). Among these, *MYBPC3* mutations often show age-dependent penetrance, with carriers developing disease later in life, whereas *MYH7* mutations are associated with earlier onset and more severe hypertrophy.

Patients harboring pathogenic or likely pathogenic variants often show higher cardiovascular mortality, increased stroke risk, greater heart failure progression, and elevated SCD risk (Bonaventura et al., 2021). Although rare (∼2.8%), compound or double heterozygous mutations are linked to more severe left ventricular dysfunction and increased heart failure risk, suggesting a possible gene-dose effect.

Beyond classical sarcomeric variants, epigenetic and environmental modifiers play crucial roles in shaping disease expression. DNA methylation patterns, microRNA regulation, and external factors such as hypertension, obesity, diabetes, renal dysfunction, and physical activity all modulate phenotypic expression and disease progression. Together, these elements underscore that HCM is not merely a single-gene disorder but a multifactorial disease where genetic, epigenetic, hormonal, and environmental factors interact to define individual risk and prognosis.

## 13 Therapies

Pharmacological therapy of HCM remains the cornerstone of symptom management across all ages (Argirò et al., 2025; Dicorato et al., 2025). β-blockers, including atenolol and nadolol, are first-line agents that reduce heart rate, myocardial contractility, and improve diastolic filling, while non-dihydropyridine calcium channel blockers such as verapamil and diltiazem serve as second-line therapy when β-blockers are ineffective or contraindicated. Persistent symptoms may be treated with the Class 1A antiarrhythmic disopyramide, and targeted myosin modulators such as mavacamten and aficamten have emerged to limit myosin–actin cross-bridge formation, reduce LVOT gradients, and improve outcomes. Gene-based interventions, though experimental, hold potential to modify the underlying genetic substrate, and invasive strategies including septal reduction surgery or alcohol septal ablation are indicated in severe or refractory cases (Argirò et al., 2025; Sikand et al., 2025; Dicorato et al., 2025; Coylewright et al., 2024). Pediatric HCM requires consideration of body size–adjusted ventricular wall thickness (Lipshultz et al., 2019) and disease heterogeneity, from rapidly progressive early-onset forms to milder adult-like phenotypes (Argirò et al., 2025; Arbelo et al., 2023). Most cases are genetic, and cause-specific diagnosis is increasingly relevant given therapies such as α-glucosidase replacement or gene transfer for Pompe disease, dasatinib and trametinib for Noonan syndrome–associated HCM, and mavacamten (Colella and Mingozzi, 2019; Yi et al., 2016; Mussa et al., 2021; Andelfinger et al., 2019; Ho et al., 2020; Spertus et al., 2021). Genotype-positive, phenotype-negative children and first-degree relatives require echocardiographic surveillance every 1–2 years in adolescence and every 3–5 years in adulthood, with early evidence supporting diltiazem or valsartan in selected cases (Ommen et al., 2020; Ho et al., 2017; Ho et al., 2021; Ho et al., 2015; Ho et al., 2016). Symptomatic management focuses on reducing LVOT obstruction via β-blockers and calcium channel blockers, with limited pediatric experience for disopyramide (Maron et al., 2003; Bogle et al., 2023).

## 14 Concluding remarks

This review provides a systematic synthesis of advances in the genetics and clinical research of hypertrophic cardiomyopathy (HCM). Its main contributions include: First, it constructs a multidimensional knowledge framework encompassing the pathogenic gene spectrum, genotype–phenotype correlations, population heterogeneity, and pediatric characteristics, thereby updating the comprehensive understanding of HCM complexity. Second, through cross-population comparisons, it reveals systematic differences in clinical phenotypes, genetic testing, and treatment patterns between Asian and European patients, while clarifying distinct genetic features in specific populations (such as the United States, Indian, Brazilian, Icelandic, Japanese, Vietnamese, South African, Finnish, and Egyptian cohorts), thereby deepening the understanding of disease specificity across populations. Third, it systematically outlines the unique aspects of pediatric HCM in terms of etiology, clinical presentation, and prognosis, offering a basis for precise management of this subgroup. Fourth, by summarizing the risk profiles associated with different genotypes, it provides direct references for clinical risk stratification, family screening, and individualized interventions, while also identifying current challenges and future research directions. This review offers a significant theoretical foundation and knowledge base for advancing HCM from generalized understanding toward precision medicine practice.

Despite significant progress, the path toward precision medicine in HCM faces several persistent challenges. The clinical interpretation of variants of uncertain significance remains a major dilemma, necessitating functional validation and larger population datasets for definitive classification. Furthermore, the considerable phenotypic heterogeneity observed even among carriers of identical mutations underscores the influence of undiscovered genetic modifiers, epigenetic regulation, and environmental factors, whose complex interactions warrant deeper investigation. While targeted therapies such as myosin inhibitors represent promising advances, translating genetic insights into effective, individualized treatment strategies remains an ongoing endeavor. Ultimately, the development of robust risk prediction models through the integration of genetic, clinical imaging, biomarker, and multi-omics data is crucial for enhancing prognostic accuracy and realizing the full potential of precision care in HCM.

Future research should focus on elucidating the mechanisms of phenotypic modulation through large prospective cohorts and novel technologies (e.g., multi-omics), advancing the clinical interpretation of variants of uncertain significance, and exploring targeted therapies for specific molecular pathways. The ultimate goal is to achieve truly personalized management of HCM, optimizing patient outcomes.
